# Supplementary material for: Are carbon emissions trading and green financial instruments synergistic? -Comprehensive quantitative research based on content analysis
Source: PLoS One. 2024 Mar 7;19(3):e0298601. doi: 10.1371/journal.pone.0298601 (PMC10919601; doi:10.1371/journal.pone.0298601)
Supplement: S1 Data — (ZIP) [file pone.0298601.s001.zip › Data/地级市Z_F工作报告.zip!/║┌┴·╜¡/╣■╢√▒⌡╩╨/└┤╫╘╣1⁄2╓┌║┼í╛┬φ┐╦╩2╛▌═°í┐_2008.docx]

各位代表：

　　我代表市人民政府，向大会作工作报告，请予审议。并请市政协各位委员提出意见。

　　一、2007年工作回顾

　　2007年，市政府在省委、省政府和市委领导下，紧紧依靠全市人民，高举中国特色社会主义伟大旗帜，以邓小平理论和“三个代表”重要思想为指导，认真贯彻落实科学发展观，深入实施东北老工业基地振兴战略，按照省委提出的哈尔滨要率先发展、率先突破、率先跨越的要求，解放思想，开拓创新，扎实工作，全力推动经济社会又好又快发展，努力促进社会和谐，完成了市十三届人大一次会议确定的各项任务，在建设“三个适宜”现代文明城市和全面建设小康社会的道路上迈出了坚实的步伐。

　　（一）国民经济好中求快，发展速度和质量持续提高

　　经济发展提速增效。紧紧扭住发展第一要务，推动速度、质量、效益协调增长，老工业基地调整改造开始显效，国民经济保持良好发展态势，主要经济指标实现新突破，登上新台阶。预计（下同）全年实现生产总值2400亿元以上，比上年增长13.5%左右，其中第一、二、三产业分别增长6%、15.5%和14.5%；人均生产总值突破3000美元，增长13%；固定资产投资突破1000亿元，增长27%；社会消费品零售总额突破1000亿元，增长15%；财政一般预算收入突破200亿元，按可比口径增长16.9%，其中地方财政一般预算收入127亿元，增长15%；进出口总额近30亿美元，增长20%，其中出口15亿美元，增长30%；规模以上工业企业实现利税135亿元，增长50%以上；规模以上工业经济效益综合指数达到160，提高近20点。

　　经济结构更趋协调优化。支持、鼓励、引导非公有制经济发展，非公有制经济占全市生产总值比重达到50%。注重经济增长的协调性，三次产业结构更趋优化，二、三产业比重明显增加。坚持把解决“三农”问题作为重中之重，积极发展现代农业，扎实推进新农村建设。实现农林牧渔业总产值583亿元，增长6%；粮食生产能力稳步提高，总产量达到97.5亿公斤，大灾之年实现保产增收；农业产业化经营机制不断完善，龙头企业竞争力明显增强。实施“工业强市”战略取得新进展。实现工业增加值近700 亿元，增长17%左右；装备制造、医药、食品和石化四个优势产业增加值，占规模以上工业增加值比重超过80%。8个工业品牌被评为中国驰名商标。大力发展现代服务业。实现旅游总收入200亿元，增长18%，在全国十佳避暑旅游城市评选中，哈尔滨市名列第3位，太阳岛风景区被评为全国首批5A级景区；成功举办了“哈洽会”、“糖酒会”、“制博会”等90余个展会；一些国内外金融机构已经或筹备在哈尔滨市设立分支机构；龙运、哈东等物流园区具备一定规模。

　　改革开放活力增强。完成了东北轻合金有限公司、中庆燃气有限公司等重点企业战略性重组。启动厂办大集体企业改制试点及供热、供排水、公交等公益性行业改革。深化财政体制改革，市本级国库集中支付制度改革实现全覆盖。新批外商投资企业99家，引进超千万美元项目18个。实际使用外商直接投资4.4亿美元，国内区域经济合作到位资金207亿元，分别增长18%和32%。世界500强在哈尔滨市投资设立企业达到20家。进出口商品结构进一步优化，机电产品、高新技术产品出口比重达到47%。企业境外投资、对外承包工程及劳务合作取得较大进展。完成对俄进出口2亿美元，增长50%。

　　新增长点带动显现。提高投资强度，改进融资方式，完善大项目管理机制，88个重点项目进展顺利。集中主要力量和优势资源推动大企业发展，促进已改制大企业释放潜能，23户大企业集团产值占规模以上工业总产值的55%。哈大齐工业走廊哈尔滨段完成投资超过28亿元。开发区进一步发展，松北、呼兰、阿城等新区焕发新的活力。

　　科技创新力度加大。实施技术创新引导工程，共投入科技创新引导资金 1.4亿元，落实产学研项目40个，确定重点技术创新项目30个，发明专利申请量增长30%，实现技术合同交易额30亿元，增长120%。推动科技创新载体和服务平台建设，新建工程技术研究中心4家，新增科技企业孵化面积2万平方米。促进创新成果产业化，59个对俄科技合作项目进入研发及产业化阶段。

　　节能减排取得成效。实施燃煤锅炉改造、热电联产等十大重点节能工程。规模以上工业企业万元工业增加值综合能耗降低6%。工业固体废弃物利用率达到76.6%。工业用水重复利用率达75%以上。完成污染减排项目605个，减排水、气污染物0.53万吨和1.5万吨。依法限期治理超标排放企业135家，关停违法排污企业35家。

　　（二）加大城乡基础设施和生态建设力度，环境承载力和吸引力持续增强

　　城乡规划工作有新进展。完善城市总体规划，完成10余项控详及重点规划，城乡规划体系基本形成。加强规划实施管理和调控，配建廉租住房、公交始末站和公厕等配套设施取得成效。加强规划联合执法，治理私建滥建和乱设户外牌匾广告初见成效。

　　城乡重大基础设施建设有新突破。哈西、群力等区域开发建设整体推进，友谊路地下通道、三环路哈阿立交桥主桥、中华巴洛克前期保护更新主体工程如期竣工，三环路江南段、道外二十道街跨江桥和何家沟污水截流工程等项目开始启动。全面完成磨盘山一期供水工程，二期供水工程的净水间、清水池等项目按计划推进。新建城区堤防42.45公里。新建改造主干道路28条，维修道路512条（段）、桥梁75座。新建农村公路3233公里，高等级路面通乡、通村率分别达到87%和55%。完成农村饮水安全工程366处，其中新建和完善老区引水工程200余处。新建沼气池9890个。

　　城市管理水平有新提升。加强城市精细化管理，加大环境综合整治力度，重点区域、背街背巷、居民庭院环境卫生质量明显改善。数字化城管体系实现中心城区全覆盖。大型公益性灯饰亮化实现统一控制。通过综合执法，整治乱贴乱画、乱停乱摆等街头顽症收到实效。城区公交编码线路和轮渡航线全部启动公交IC卡系统。

　　生态建设和环境保护有新加强。积极创建国家园林城市和环保模范城市，实施生态恢复工程建设，治理水土流失50万亩、草原“三化”5万亩，植树造林13.5万亩，新增绿化面积1.05万亩。实施松花江流域治理工程，市区污水集中处理率达到42%，集中供热普及率达到63%，市区空气质量基本达到国家二级标准。在首届中国最美丽城市排行榜中，哈尔滨市位居第20位。

　　（三）稳步推进社会建设，人民生活持续改善

　　社会事业进一步发展。努力促进教育公平和均衡发展，落实农村义务教育经费保障机制，全部免除农村义务教育阶段学杂费，6万名外来务工人员子女与城市学生享受同等教育。改善办学条件，建设寄宿制学校13所，改造薄弱学校22所。完成哈尔滨技师学院一期工程建设，并投入使用。加快文化建设，哈尔滨艺术中心、市博物馆建设顺利启动，以流动图书馆、演出服务网为重点的公共文化服务体系建设成效显著，10项民俗文化入选首批省级非物质文化遗产名录。推动公共卫生体系建设，社区卫生服务网络进一步完善，农村常住人口参合率达到94.1%。人口和计划生育工作进一步加强，人口自然增长率控制在4.4‰以下。竞技体育成效显著，群众性体育活动蓬勃开展，体育民间组织快速发展，第24届哈尔滨世界大学生冬季运动会场馆设施建设和各项筹备工作进展顺利。

　　就业和社会保障水平进一步提升。鼓励创业带动就业，发展小企业和个体工商户1.7万家。全市城镇新增就业近10万人，安置零就业家庭就业9700多人，登记失业率控制在3.5%以内。转移农村劳动力120万人。全面启动城镇居民医疗保险，实施困难企业退休职工医疗保险，扩大了城镇职工养老保险、失业保险和医疗保险覆盖面。提高城镇居民最低生活保障标准，实现应保尽保。建立农村低保制度，纳入农村低保11.6万人。初步建立农民工工资保障制度。投入9113万元，对56.6万救助对象进行分类救助。

　　城乡人民生活水平进一步提高。城市居民人均可支配收入超过12600元，增长13%；农民人均纯收入突破5000元，增长15%。扩大廉租住房受益面，解决廉租住房2056户，是前四年总和的1.26倍，实现人均7平方米以下廉租户应保尽保。出台新的拆迁补偿办法，完成40余万平方米危棚房拆迁任务。发展普惠型公益事业，完成天然气置换前期准备工作，城区124.8万人饮用磨盘山优质水，农村居民饮用洁净水超过60%。开辟延伸农村客运线路33条，客运班车通村率达到97.5%。加强食品、药品安全监管和市场调控，保障食品、药品安全和市场供应。投入新农村建设资金21亿元，用于改善农村生产生活条件，发展农村社会事业。惠民行动的20件实事全部落实。

　　（四）加强精神文明和民主法制建设，思想道德和公共服务水平持续提高

　　精神文明建设扎实推进。深入开展社会主义荣辱观教育，全市人民的思想道德水平进一步提高。深入开展“和谐社区、和谐单位、和谐校园、和谐机关”活动，和谐基础创建活动效果明显。加强国防教育、国防后备力量建设，圆满完成征兵工作。荣获东北三省“双支”活动模范单位称号。

　　民主法制建设全面加强。自觉接受人大法律监督和政协民主监督，人大代表建议、政协委员提案办复率100%。征集人民建议4300件。坚持依法行政，完善行政执法责任制，创新行政复议工作机制，依法解决社会矛盾的能力进一步增强。全年提请市人大常委会审议地方性法规草案2件，制定政府规章24件。实施“五五普法”，社区矫正工作开始起步。依法完成了第二届社区居委会换届选举工作。加快“平安哈尔滨”建设，社会治安防控体系逐步健全，刑事案件发案率下降2.4%。加大各类安全隐患整改和治理力度，重特大安全事故得到有效遏制。在首届中国最安全城市排行榜中位居第15位。坚持领导干部包案和信访接待日制度，及时处理了一批重点疑难信访案件，进京、到省非正常访分别下降11.3%和6.8%。社会管理和公共服务得到加强。围绕服务型政府建设，把政府工作、政策支持和公共财力保障的重点，向健全公共服务体系、增加公共产品和发展社会事业倾斜。优化政务环境，行政审批环节进一步简化。修订59项专项应急预案，应对和处置突发公共事件机制更加完善。推进政务公开，提高政府工作透明度，扩大群众的知情权和参与权。

　　妇女儿童、新闻出版、广播电视、民族宗教、人事、统计、审计、外事、侨务、对台、老龄、气象、人防、地震、档案、地方志和残疾人等项工作也取得长足进步。

　　各位代表，新一届政府开局之年取得的成绩，是市委正确领导的结果，是人大、政协及各界人士大力支持的结果，是全体市民群众共同努力的结果，是国家、省及中省直驻哈企事业单位以及社会各界配合帮助的结果。在此，我代表市人民政府，向全市广大工人、农民、知识分子和干部，向各民主党派、工商联、无党派人士和社会团体，向驻哈人民解放军、武警官兵和公安干警，向所有关心、支持哈尔滨发展的同志们、朋友们和海内外友好人士，表示崇高的敬意和衷心的感谢！

　　在肯定成绩的同时，我们也清醒地认识到经济社会发展中还存在一些突出问题，政府工作及自身建设仍有不少差距。一是经济发展与同类城市的差距仍在扩大，速度不够快、质量不够高、效益不够好、结构不够优的问题比较突出；二是改革开放差距仍然很大，市场化和开放程度明显偏低；三是城乡基础设施建设欠账较多，发展环境的竞争优势不够突出；四是社会事业和社会保障相对薄弱，公共产品和服务供给能力不足；五是“三农”问题仍比较突出，县域经济基础比较薄弱，农业现代化、产业化和农村工业化、城镇化水平仍然较低，新农村建设任务艰巨；六是社会不稳定、不和谐因素还比较多，维护稳定和处理突发事件的压力仍然较大；七是安全生产事故尚处于易发多发期，一些令人痛心的安全责任事故还时有发生；八是一些政府工作人员精神不振、推诿扯皮、奢侈浪费和腐败现象仍然存在，政府自身建设尤其是效能、廉政建设任务十分艰巨。对上述问题，我们在新一年工作中将继续着力加以解决。

　　二、2008年工作安排

　　2008年是我国改革开放30周年，是全面贯彻落实党的十七大战略部署的第一年，是国家实施《东北地区振兴规划》的开局之年，也是哈尔滨市实施“十一五”规划承上启下之年和全面完成第24届哈尔滨世界大学生冬季运动会筹备工作的关键之年。我们要高举中国特色社会主义伟大旗帜，全面贯彻党的十七大精神，坚持科学发展、和谐发展，坚持好中求快、稳中求进，更加坚定自觉地推进改革开放和自主创新，更加坚定自觉地推进经济结构调整和发展方式转变，更加坚定自觉地推进区域协调发展和城乡统筹，更加坚定自觉地推进节能减排和环境保护，更加坚定自觉地推进改善民生和社会和谐，着力加强经济、政治、文化、社会和生态文明建设，不断提高城市综合竞争力，奋力创造 “三个适宜”现代文明城市建设新业绩，奋力夺取全面建设小康社会新胜利。经济社会发展的主要预期目标是：

　　———经济发展速度、质量、效益、结构进一步提升。生产总值增长12.5%，万元生产总值综合能耗降低4%，科技进步对经济增长的贡献率达到49%以上，地方财政一般预算收入按可比口径增长12.5%，全社会固定资产投资增长25%，社会消费品零售总额增长13%，市属进出口总额增长15%，实际直接使用外资额增长18%，国内区域经济合作到位资金增长30%。

　　———生态和环境质量进一步提高。森林覆盖率达到44.6%，治理水土流失50万亩，治理草原沙化、碱化和退化6万亩，水、气主要污染物总量分别削减3%和1%。

　　———基础设施和城市建设进一步加快。以重点项目建设为牵动，城市载体功能和农村生产生活环境有较大提升；以危房棚户区改造为重点，市民群众住房条件有较大改善；以筹办大冬会为契机，促进城市环境形象有较大改观。

　　———社会事业、人民生活进一步改善。城市居民人均可支配收入增长8%，农村居民人均纯收入增长6%，城镇登记失业率控制在4.6%以内，社会保障覆盖面进一步扩大，教育、卫生、文化等社会事业有较大发展，人口自然增长率控制在4.5‰以内。

　　为实现上述目标，重点抓好10项工作。

　　（一）推进社会主义新农村建设

　　加强农业和农村基础设施建设。制定农田水利基础设施建设五年规划，重点推进5个大型灌区续建配套和节水改造，启动5个沿江提水灌区资源整合工程。继续推进农村饮水安全工程建设。新建农村公路1500公里、乡镇客运站10个，开辟延伸客运线路10条，行政村全部开通客车。新建大中型沼气工程20处，发展户用沼气池1.29万个。完成农村康居工程200万平方米。继续搞好新农村建设试点工作。积极稳妥推进城镇化，加快40个区域中心镇建设。实施70个村扶贫开发整村推进。

　　加快发展现代农业。用现代物质条件和先进适用技术武装、改造农业，提高农业综合生产能力。加快建设国家优质粮食生产核心区，谋划建设国家级农业科技示范区。推进农业机械化、标准化和良种化。深入实施科技入户工程和“三电合一”工程，抓好20个农业信息化示范村建设。加大中低产田改造力度，改造中低产田25万亩。突出区域特色，鼓励发展“一村一品、一乡一业”。规划提升水稻、玉米、大豆等8个优势产业带，无公害、绿色和有机农产品认定面积扩大到2600万亩。推进畜牧业规模化养殖，强化动物疫病防控和畜产品安全设施建设。强化农业气象灾害监测预警，加快防汛抗旱指挥系统建设，提高防灾减灾能力。

　　加速推进农业产业化。以农业产业化带动农村新型工业化，做大做强粮、乳、肉、酒等食品工业，培育壮大龙头企业，拉长乳品、生猪、肉牛等8个产业链。推进4个国家级绿色食品原料标准化生产基地建设，打造五常大米、大众红肠、雪莲鸡肉等20个绿色食品知名品牌，建设东北重要绿色食品产业集聚区。大力发展农村新型合作经济组织，提高农民组织化程度。

　　继续推进农村改革。全面落实惠农政策，及时发放各种涉农补贴资金。深化乡镇机构改革，提高服务水平和行政效率。完善“乡财县管”和“村财乡理”办法。稳定和完善土地承包关系，发展多种形式的适度规模经营。加强农村民主政治建设，依法组织第8届村民委员会换届，做好农村社区建设试点工作。按照“谁投资、谁受益、谁所有”的原则，加快小型农田水利工程建设管理体制改革。启动水权水市场改革。探索农村集体经济的有效实现形式。搞好村镇银行试点，推进林权制度改革试点。

　　提高农村劳动者素质。积极开展多种形式的农民职业教育和农业生产技能培训，培育一批有文化、懂技术、会经营的农村致富带头人，培训农村劳动力35万人，提高农民的科学务农水平，增强自我发展能力。

　　（二）推进新型工业化进程

　　以大项目为牵动，培育壮大优势产业集群和大企业集团。促进要素资源向优势产业、骨干企业集中，推进装备制造、医药、食品、石化等优势产业集群发展，延伸产业链条，打造汽车、电站成套设备、农副产品深加工、抗生素类药物、精细化工等产业链。以项目建设支撑产业链条延伸、促进产业配套。实施投资超五千万元项目100个、超亿元项目50个，年内竣工投产50个，工业固定资产投资增长25%以上。以装备制造、医药、食品和石化四大优势产业为骨干，继续提高产业集中度。以发展新能源、新材料、生物制药等高新技术产业为方向，加快资源整合，启动风电产业制造基地等项目建设。改造提升冶金、轻纺、建材等传统产业。强化激励机制，对贡献大、效益好、发展快的企业及相关人员实行重奖。

　　以工业园区为依托，调整优化工业布局。按照工业布局调整方向，扩容壮大一批、改造提升一批、优化整合一批工业园区。建设以机电、汽车等为重点的装备制造业集聚区，壮大医药食品集中区，改造提升化工园区，打造高新技术产业板块，整体提高县域工业园区的规模和实力。全面推进哈大齐工业走廊哈尔滨段启动区基础设施建设，新增协议入区项目70个，协议投资50亿元，建成投产50个。制定工业布局导向政策，从土地、税收、资金等方面支持布局优化调整，有序推进老企业向专业园区搬迁集聚。

　　以企业信息化为先导，提升工业整体素质。以装备制造业为重点，推动企业信息化。引导中小企业广泛应用信息技术。鼓励高新技术企业开发数字化产品。依托企业技术中心、工程中心和中介机构，建立企业信息化服务联盟。推动虚拟企业公共服务平台建设。

　　（三）推进服务业优化升级

　　提升商贸服务业。继续推进城乡商品市场体系建设，不断优化中心区、新城区、郊区和农村商业布局。鼓励发展经营业态多样化的便民服务业。积极发展连锁经营、特许经营。持续推进“万村千乡市场工程”。健全生活必需品市场和重点流通企业监测系统，完善生活必需品政府储备制度和市场应急预案。

　　发展旅游会展业。围绕打造“冰城夏都”旅游胜地，完善旅游发展规划。加强旅游基础设施建设，深度开发旅游产品，放大冰雪旅游、避暑旅游、生态旅游等品牌效应。配合全国总工会，推进以劳模主题公园为重点的项目建设。积极发展森林旅游、工业旅游和农业旅游。实现接待国内外游客增长13%以上，旅游总收入增长17%以上。制定完善会展业发展规划和相关配套政策，积极引进国内外大型展会，高质量筹办好第19届“哈洽会”、第63届“汽博会”、第8届“制博会”、第2届“哈科会”等大型展会和节庆活动。

　　壮大现代物流业。实施现代物流业发展规划，推进公路总枢纽、新香坊集装箱节点站等大型物流项目和龙运、神州等物流园区建设，发展第三方物流，建设辐射全省、全国乃至连接国际市场的物流平台。

　　拓展现代金融业。推动市商业银行在省外设立分支机构、江海证券实现综合性经营、中融信托引进战略投资者。争取更多国内外银行在哈尔滨市设立机构，吸引更多的证券公司、保险公司、信托公司进入哈尔滨市。支持企业在境内外上市融资。打造群力金融商务区。完善社会信用体系，优化金融生态环境。

　　培育现代信息和中介服务业。加快发展软件业，发展电子商务。完善服务外包产业发展规划和扶持政策，培育服务外包企业群体和大企业。完成服务外包基地一期工程建设。规范发展会计、律师、公证、投资咨询、资产评估、信用评级等中介服务业。

　　（四）推进改革开放

　　继续深化各项改革，着力构建又好又快发展的动力机制和体制保障。扎实推进国家在哈尔滨市进行的各项改革试点工作。基本完成具备改制条件的厂办大集体企业改制任务。继续深化城镇居民医疗保险、户籍制度改革试点工作。加快市场化进程。继续推进供水、供热、公共客运等公用公益事业企业改革，推动哈工业资产经营公司进行战略性整体重组，构建市级国有资产投融资平台，打造百亿规模的产业集团。积极推进企业债务重组，力争回购债权10亿元以上。深入推进国有企业主辅分离，基本完成市属国有企业家属区的供水、供热及物业分离。妥善处理国企改制遗留问题。推进20户国家已批准政策性破产项目实施。积极发展各类生产要素市场。继续深化各项制度改革。加强国有资产监管，规范产权、股权转让和交易行为，推行国有资本经营预算制度，落实国有企业重大投融资项目报告和审批制度，在工业资产经营公司、物业供热集团、供排水集团等企业开展经营业绩考核试点。开展向国有大中型企业派驻监事会或监事的试点工作。完善公共财政制度，调整支出结构，较大幅度增加对社会保障、卫生、教育、住房保障等方面的支出。推进政府采购制度改革。扩大财政支出绩效评价试点和参与式预算试点。推行项目公示制度，扩大代建制试点，完善项目谋划生成、推进协调、监督服务和考核奖惩机制。实行“在地统计”，推动“区、县（市）属经济”向“区域经济”转变。

　　继续扩大全方位开放，着力提高开放型经济水平。推动外贸经营主体多元化，重点扶持民营企业和外贸龙头企业做大做强。在继续巩固美、日、韩、俄、欧盟等传统市场的同时，大力开发东盟、中东、南美和非洲等新兴市场。优化进出口商品结构，鼓励战略性资源和高耗能商品进口，支持汽车、医药、电子产品、电站成套设备、深加工农副产品出口。继续加强对俄对韩经济贸易、资源合作利用、高技术引进和产业化工作，推动对俄对韩经贸科技合作战略升级。发展承接离岸外包业务。坚持“引进来”与“走出去”相结合，支持优势企业到境外建立贸易公司、生产基地和研发中心，拓展劳务输出、技术合作、工程承包等合作新领域。进一步拓宽国内外区域经济合作领域，除国家明令禁止的，一律向境内外投资商开放。完善招商项目生成机制，创新招商引资方式，更多地吸引外来资金。以具有牵动力的大项目和高效服务的行政环境吸引战略投资者，推动产业升级和重大基础设施建设。以优惠政策支持外商到哈尔滨市设立区域总部、研发中心、采购中心和培训中心。完善招商引资责任、激励和重点项目服务机制，对投资超千万美元或超亿元人民币的大项目，实行市与区、县（市）两级领导包保责任制，切实提高招商项目履约率、资金到位率和项目开工率。

　　（五）推进创新创业型城市建设

　　完善科技创新服务体系平台。持续推进以企业为主体、产学研相结合为途径、产业技术为重点的技术创新体系建设；以高等院校和科研院所为依托的技术原始创新体系建设；社会化、网络化为载体的科技中介服务体系建设。持续推进技术研发、科技资源信息共享、科技企业孵化、技术交易和科技投融资五个科技创新平台建设。建立健全激励自主创新的人才评价和奖励制度。推进国家知识产权示范城市试点，发挥国有企业在自主创新中的重要作用，大力实施知识产权战略，实现专利申请量3900件以上，支持拥有自主知识产权的产品实现产业化。鼓励和支持科技型中小企业加快发展。

　　支持重点科研攻关。加大财政投入，发挥企业科技创新主体作用，重点推进百万千瓦等级核岛、国产碳纤维复合材料及应用、风力发电、生物制药及农作物优质抗逆新品种培育等关键技术研发。完善地理信息产业和动漫产业公共技术平台。支持重点行业和骨干企业建设一批市级以上工程研究中心和企业技术中心。实现高新技术产业产值增长20%以上。进一步做好科普工作。

　　深入推进全民创业。制定全民创业发展规划，加快培育创业主体。制定相关政策，引导高校毕业生自主创业和灵活就业，鼓励和推动农民创业，支持各类企业实现再创业。推进公平准入，改善融资条件，降低创业门槛，增加创业培训投入，促进个体、私营经济和中小企业发展，使更多的劳动者成为创业者。充实民营企业、中小企业信用担保和“小巨人”企业担保基金，为民营中小企业创业提供担保支持。

　　（六）推进宜居城市建设

　　完善城乡规划。进一步完善城乡规划编制体系，组织编制《印象哈尔滨专题规划》、《危棚房改造规划》、《防灾疏散空间规划》、《公共服务建设规划》、《新区建筑风格规划》、《市域村镇体系规划》。完善规划管理、监督体系和科技手段，进一步提高规划工作水平。继续加强“联合执法”，严肃查处开发违规违法行为。

　　继续加大城市基础设施建设力度。实施道外二十道街松花江大桥、磨盘山供水二期、“三马地区”改造、何家沟综合整治、西泉眼水库维修加固工程。启动何家沟群力污水处理厂、平房污水处理厂、西南部集中供热、华能热源扩建、内河清水水源建设工程。启动三环西桥、轨道交通一期工程建设。实施哈尔滨火车站综合改造和外延性道路打通工程。配合铁道部推进哈西火车站和哈大客运铁路建设。根据大顶子山航电枢纽工程带来的水位变化，加快推进沿江堤防工程建设。实施路畅工程，推动城区路网全面升级改造。继续推进行人过街桥、无障碍通道、公厕等便民设施建设。加强公交基础设施建设，新增、更新公交车辆120台。

　　推进城市环境综合整治。采取市区结合、以区为主的方式，以贴近民生的环境整治项目为重点，改造建设500个居民庭院、50个社区休闲健身广场，同步改造周边破损道路，解决居民出行难问题。推进灯饰亮化工程，丰富城市夜色景观。加强历史文化名城保护，传承历史文脉，打造特色景观。实施秋林商务区等改造工程，提升中心城区环境形象。进一步加大交通环境整治力度，严格查处交通违规违法行为，促进城市交通状况明显好转。实施松花江哈尔滨段综合治理工程，建设沿江景观带，重点搞好金河湾湿地植物园等整治工程，建设滩涂生态景观区。推进临江建筑节能性景观改造。围绕奥运火炬传递活动和举办大冬会，实施赛场、运动员村周边环境净化、绿化、美化、亮化工程，进一步推进哈阿出城口和奥运火炬传递线路周边环境综合整治。

　　加强城市管理。按照创建国家园林城市、国家卫生城市要求，进一步整治外部建筑物和设施蒙尘污渍，乱扔乱倒、乱停乱摆、乱贴乱画等不文明现象。合理划分市、区事权财权，落实管理责任。加强街道办事处公共服务职能，推进城市管理进社区。实施“数字城管”网络三期工程，完善快速反应和及时处置系统。实行标准化检查考核，建立科学合理的城市管理考评体系。按照“精心规划、精制建设、精细管理”要求，推进精细化管理长效机制建设。加强城区科学绿化和管护，提高绿化水平和成活率。新增绿地860公顷，新植树木110万株。采取切实措施，提高城市环境卫生管理水平。

　　深化城市经营实践。强化政府对土地一级市场培育、开发和管理，收回超期未开发的闲置土地，最大限度地盘活城市存量土地资源。鼓励支持社会力量投资建设公园、路桥、供水和垃圾处理等基础设施，大力推动清冰雪、环卫保洁、市政设施维护、园林绿化养护等公用事业市场化运作，面向社会有偿转让停车场、空间广告、公交线路和公共设施冠名等公共资源经营管理权。采取建设-经营-移交、资产证券化、委托借款等模式，解决城市基础设施和公用事业项目建设资金问题。编制沿江水利产业带规划，组建水务投资公司，科学利用水资源，发展水经济。

　　（七）推进资源节约型和环境友好型城市建设

　　加大节能减排力度。增加节能减排资金投入。严格控制高耗能、高污染新建项目和规模以上工业企业燃煤增量。着力抓好电力企业二氧化硫减排，限制能耗高、污染重机组发电。关停200兆瓦小火电机组，淘汰10户企业落后产能。加快分散供热锅炉联片并网改造，淘汰50台7兆瓦以下燃煤供热锅炉。完成松花江污染防治和二氧化硫治理工程项目20个。实施马家沟污水转输、文昌污水处理厂污泥综合利用、沿岸重点排污企业水污染源治理工程。建立科学、完整、统一的节能减排统计、监测和考核体系，把节能减排纳入政府领导工作评价和企业负责人业绩考核内容，实行严格的问责制。深入开展节能减排全民行动。单位工业增加值综合能耗下降4%。

　　大力发展循环经济。推进工业三废综合利用等6个产业化示范工程。建立危害环境的废弃物回收处理产业化体系，工业固体废弃物综合利用率提高1.2个百分点，达到77.8%。开发风能、太阳能和地热等可再生资源，建设依兰、方正等3个风力发电场项目，新建、改建3个热电联产项目。启动建设市开发区和利民开发区两个循环经济专业园区。

　　加强生态环境建设和保护。推进松花江干流、一级支流水域环境综合整治。实施天然林保护等生态恢复工程，造林绿化10万亩，建设省级以上自然保护区2个。启动实施国家一期黑土地治理工程。实施《哈尔滨市农村小康环保行动计划》，探索水行政执法新机制，在饮用水源地一级保护区、松花江流域干支流两侧1公里范围内村屯，实行畜禽粪便、污水、垃圾综合整治，对施用农药、化肥实行严格监控。建立重大污染事故快速反应机制，完善环境与发展综合决策机制。推行有利于环境的生产和消费方式，创建环境友好型企业、环保宜居社区、绿色学校、环境优美乡镇、生态文明示范村100个。

　　（八）推进文化大发展大繁荣

　　培育城市文明风尚。深入开展中国特色社会主义理论宣传普及活动，加强以“八荣八耻”为主要内容的社会主义荣辱观教育，把社会主义核心价值体系融入市民教育和精神文明建设全过程。大力弘扬爱国主义、集体主义、社会主义思想和开放包容、时尚活力、诚信敬业、和谐奋进的城市精神，加强社会公德、职业道德、家庭美德、个人品德建设。

　　发展公益性文化事业。加大财政对公益性文化事业投入，广泛动员社会力量，加快建设覆盖城乡的公共文化服务体系，切实保障人民群众基本文化权益。建设市博物馆，改建部分市级文化场馆，新建一批社区图书馆。加强农村文化设施建设，建设和完善一批农村文化站。注重丰富进城务工人员的精神文化生活。加强文化人才引进、培养和使用工作，最大限度地激发文化创造活力，创作更多的精神文化产品。发展群众性文化娱乐项目。加强本地区非物质文化遗产保护。高质量办好第29届“哈夏音乐会”。

　　不断壮大文化产业。推进文化体制机制创新，积极发展新闻出版、文化娱乐、电影音像、艺术演出等传统文化产业，大力培育文化创意、文化博览、文化旅游、文化节庆、数字电视、网络传输等新兴文化产业。支持黑龙江动漫产业（平房）发展基地做大做强。推进文化市场存量资源整合升级。严肃查处违法音像制品经营活动，净化媒体宣传内容，坚决取缔黑网吧。

　　（九）推进大冬会各项筹备工作

　　加快大冬会场馆设施建设。按时完成市冰球馆、市滑冰馆和亚布力滑雪场等基础设施建设。规划建设全民健身中心，改造八区体育运动中心。

　　完善大冬会服务保障系统。加强商业、餐饮、旅游等窗口行业定向培训，提高从业人员技能和服务水平。促进大冬会筹办工作向赛时转变。围绕治安、交通、市场、环卫、旅游等领域，建立大冬会期间城市运行指挥体系，搭建城市运行控制平台。大力普及公共安全知识，开展综合应急演练。做好大冬会志愿者招募和培训工作。

　　营造大冬会社会氛围。开展“迎奥运、迎大冬、讲文明、树新风”活动。抓好市民文明礼仪教育和外语培训，培养市民遵规章、守秩序的良好习惯。深入开展全民健身活动。加强青少年优秀体育后备人才基地建设，推进机关、学校、企事业单位体育设施向社会开放。做大体育产业，带动相关产业发展。

　　（十）推进社会事业发展和民生改善

　　推动教育均衡发展。继续加大教育投入，全面实施城乡免费义务教育。深化课程改革，推进素质教育。加大中小学校舍建设力度，新建、扩建5所中小学校，继续建设农村寄宿制学校，重点改造和更新18所学校的教学设备，维修改造18万平方米B、C级危房校舍。发挥农村中小学远程教育网作用，实现优质教育资源共享。加强教师队伍建设，重点提高农村教师素质，促进校长、教师合理流动。发展职业技术教育，培养本地经济社会适用人才。办好哈尔滨技师学院。关注特殊教育，推动全民终身教育。

　　努力扩大就业。实施扩大就业的发展战略，促进以创业带动就业，新增城镇就业8.5万人，转移农村劳动力122万人。对零就业家庭实行“托底”式安置。建立与企业利润挂钩的职工工资同步增长机制和支付保障机制，杜绝各种拖欠工资行为，形成和谐劳动关系。

　　完善覆盖城乡的社会保障制度。推动养老保险扩面，提高城镇职工基本医疗保险待遇水平，加强城镇居民医疗保险工作。探索建立农村新型养老保险制度。加大社会保险征缴和基金监管力度。逐步完善以居家养老为基础、社区为依托、机构为补充的社会福利服务体系。进一步加大养老服务机构建设，整合建成并投入使用20所农村敬老院，床位达到3000张以上；完成第一社会福利院主体工程建设；鼓励社会力量兴办老年福利机构，新增养老床位1000张，老年人口集中供养床位拥有率由11‰提高到13‰。加大市区两级投入，分步建设社区公共服务设施，构建以星光老年之家、社区服务中心（站）为依托的为老服务格局。全面启动以政府购买服务为主要方式的居家养老服务，对生活困难的独居或重病老人支付居家养老服务费用。扩大最低生活保障范围，构筑面向低保对象和低收入家庭的社会救助网络。加强城市专项救助，推进城镇低保救助体系向农村延伸，完善农村大病医疗、法律援助、劳动力输出等项救助制度。农村低保标准不低于人均800元，集中和分散供养五保对象年生活费，分别由2200元、1600元，提高到2600元、1800元。合理界定低收入家庭标准，研究制定相应的配套救助政策。完善和落实因基本生活必需品价格上涨对低收入群众的补助办法。启动“中福在线”，深入开展慈善募捐活动。建设残疾人托管服务中心。

　　加强公共医疗卫生服务。完善新型农村合作医疗制度，加强农村三级卫生网络建设，推进院前急救服务向农村延伸。坚持政府主导，加大投入，完成45个社区服务中心（站）新建或改扩建，装备71个社区医疗卫生服务中心（站）。支持大医院创办、领办社区医疗卫生服务机构，支持非市属大医院和社会医疗卫生力量，开展农村和社区医疗卫生服务，做到“小病在社区，大病进医院，康复回社区”。开展医疗卫生形象年、医院管理年活动，提高市属医院医疗服务水平。严格实施价格公示制度，探索建立常见疾病规范诊疗程序和价格体系，对单病种实行最高收费限价。加大药品集中招标采购力度，提高广大群众对医疗服务的满意度。加强疾病预防工作，完善疫情报告和疾病监测网络，加强传染病和地方病防治。发展中医中药事业。

　　改善群众住房条件。实施“好民居”计划，建立住房保障体系，增加廉租住房和经济适用住房供给量。积极调控住房价格，优化住房供给结构。扩大廉租住房实物配租比例，为人均住房使用面积8平方米以下的廉租住户解决住房困难。坚持依法和谐拆迁，全面启动三环线以内危房棚户区改造，改造危棚房100万平方米。完善拆迁补偿最低保障制度。改善建筑工人工地住宿和外来务工人员居住条件。全年竣工住宅450万平方米。

　　推进“平安哈尔滨”建设。健全党委领导、政府负责、社会协同、公众参与的维护稳定工作格局，启动建设群众诉求服务中心，建立司法救助专项基金，建立完善维稳工作领导责任制、社会管理利益诉求机制、群体性突发事件协调机制、矛盾排查调处机制和社会稳定风险预测评估机制，妥善处理各种矛盾纠纷。加强社会治安综合治理，继续深化“打黑除恶”专项斗争，严厉打击境内外敌对势力、敌对分子和“法轮功”等邪教组织的渗透破坏活动。加强治安防控体系建设，提高城乡治安防范水平。加强流动人口管理，落实对“虚拟社会”、犯罪高危人群的管理措施。高度重视生产安全、交通安全和消防安全，坚决遏制重特大安全责任事故。强化公共安全管理，建立健全覆盖全市的应急管理网络体系和应急指挥网络平台。加强隐蔽战线斗争，切实维护国家安全。加强市场监管和行政执法，强化农产品、食品、药品等质量安全标准体系建设，实行市场准入、不合格产品退市制度，着力解决制售假冒伪劣商品等危害民生的突出问题。

　　继续做好人口和计划生育、民族宗教、广播电视、新闻出版、外事侨务、对台、人防、民兵和预备役、气象、统计、档案、地方志等工作。

　　三、加强政府自身建设，提高公共服务水平

　　继续加强政府效能和作风建设，提高政府的公信力、执行力和工作透明度，提高政府服务市民群众、服务市场经济主体的能力，不断满足社会公众日益增长的公共产品与服务需求，建设行为规范、公正透明、勤政高效、清正廉洁，具有公共服务精神，让人民群众真心拥护、放心满意的政府。

　　加强服务能力建设。继续推进事权下放和管理重心下移，将事务性管理职责逐步下放给区，将大量的社会性服务事务转移给社会中介组织和城市社区。进一步理顺社区管理和服务模式，改善社区办公条件，提高经费标准，解决一批办公用房。深化行政管理体制改革，优化政府工作流程，继续精简许可事项，减少审批环节，缩短办理周期，强化审批后监督管理。按照中央和省统一部署，探索实行职能有机统一的大部门体制。改革政府工作目标生成模式，拓宽人民群众参与渠道，使政府的重大决策，特别是利民、惠民行动，更多地听取人民群众意见。开展服务对象评议服务者活动，对位列后三位部门的领导严格问责。从解决突出的社会矛盾和供求矛盾入手，增加急需和短缺的公共产品供给。搞好各级行政服务机构和政务大厅建设，加大即时即办件比重，提高审批服务“窗口”的办结率。完善服务体系，健全行政审批代办制、联审联办制、首办负责制、服务承诺制和限时办结制。推动电子政务网络平台建设，启动政府系统政务内网建设，加快信息资源开发利用。

　　加强法治政府建设。加强依法行政工作，将合法性审查的范围从出台政策法规，拓展到部门行政决策，保证政府所有的职责、权限、行政决定和行政程序有法定依据。强化行政执法责任制，完善行政复议新机制，落实行政纠错和行政赔偿制度。清理和调整没有充分体现公平、公正、便民原则的市政府规章和规范性文件，规范行政行为和行政执法自由裁量权，强化行政执法监督力度。探索在城市管理以外的管理领域和区、县（市）政府实行相对集中行使行政处罚权。主动接受人大的法律监督、政协的民主监督、公众的社会监督和媒体的舆论监督。支持工会、共青团、妇联开展工作，加大妇女儿童发展两个规划实施力度。

　　加强制度建设。进一步健全重大事项集体决策、专家咨询、社会公示与听证、决策评估等制度。强化大项目等重点工作的专项推进机制、联动互动机制、专项服务机制、科学调控机制、沟通协调机制和执行保障机制。健全政府绩效考核评价体系，在现有考评体系基础上，建立健全以改善民生为重点的社会和谐考核评价体系、政务环境评价体系。探索建立公众参与的政府绩效考核办法。优化和拓宽目标考核内容，改进目标管理方式。利用电子政务平台，研究建立市级重点工作实时监控系统。实行“阳光行政”，推行政务公开，建立健全政府发言人、政府网站、政府公报和政府短信平台等政务信息公开的制度和渠道，只要不涉及国家机密的所有政策、决策一律向社会公开，切实保障人民群众对政府工作的知情权、参与权、表达权和监督权。

　　加强廉政建设。进一步加大对企业国有产权交易、政府采购、土地出让、工程建设招投标、项目规划审批等重点领域和环节腐败行为的查处力度，持续深入地开展治理商业贿赂专项工作。坚持教育、制度和监督为内容的惩防并重方针，健全行政权力运行约束机制，实现用制度管权、按制度管事、靠制度管人，从源头上预防腐败发生。深入实施《哈尔滨市行政机关领导问责暂行办法》。降低行政成本，严格控制财政一般性支出，推进公车制度改革，杜绝铺张浪费，建设节约型政府。

　　加强作风建设。坚持科学执政、民主执政、依法执政，按照“为民、亲民、务实、清廉”的要求，提高各级领导班子战略思维、工作创新和宽思路、高起点、大手笔谋划科学发展的能力。以建设学习型政府，提高政府工作人员知识化、专业化、国际化水平为重点，加强公务员队伍建设，全面提升公务员的服务意识和业务素质，使广大公务员始终保持昂扬向上的精神状态，积极进取的工作状态，躬身为民的服务状态，树立人民公仆的良好形象。

　　各位代表，哈尔滨市明年的发展任务十分艰巨。我们要在党的十七大精神指引下，紧密团结在以胡锦涛同志为总书记的党中央周围，在省委、省政府和市委的领导下，团结依靠全市人民，坚定信心、锐意创新、奋发进取、扎实工作，为早日实现建设“三个适宜”现代文明城市和更高水平小康社会的宏伟目标而努力奋斗！
